# Progesterone reduces erectile dysfunction in sleep-deprived spontaneously hypertensive rats

**DOI:** 10.1186/1477-7827-5-7

**Published:** 2007-03-01

**Authors:** Monica L Andersen, Raquel CS Martins, Tathiana AF Alvarenga, Isabela B Antunes, Ligia A Papale, Sergio Tufik

**Affiliations:** 1 Psychobiology Department – Universidade Federal de São Paulo, Escola Paulista de Medicina (UNIFESP/EPM), R. Napoleão de Barros, 925, V. Clementino 04024-002, São Paulo, SP, Brazil

## Abstract

**Background:**

Paradoxical sleep deprivation (PSD) associated with cocaine has been shown to enhance genital reflexes (penile erection-PE and ejaculation-EJ) in Wistar rats. Since hypertension predisposes males to erectile dysfunction, the aim of the present study was to investigate the effects of PSD on genital reflexes in the spontaneously hypertensive rat (SHR) compared to the Wistar strain. We also extended our study to examine how PSD affect steroid hormone concentrations involved in genital events in both experimental models.

**Methods:**

The first experiment investigated the effects of PSD on genital reflexes of Wistar and SHR rats challenged by saline and cocaine (n = 10/group). To further examine the impact of the PSD on concentrations of sexual hormones, we performed a hormonal analysis of testosterone and progesterone in the Wistar and in SHR strains. Since after PSD progesterone concentrations decreased in the SHR compared to the Wistar PSD group we extended our study by investigating whether progesterone (25 mg/kg or 50 mg/kg) or testosterone (0.5 mg/kg or 1.0 mg/kg) administration during PSD would have a facilitator effect on the occurrence of genital reflexes in this hypertensive strain.

**Results:**

A 4-day period of PSD induced PE in 50% of the Wistar rats against 10% for the SHR. These genital reflexes was potentiated by cocaine in Wistar rats whereas this scenario did not promote significant enhancement in PE and EJ in hypertensive rats, and the percentage of SHR displaying genital reflexes still figured significantly lower than that of the Wistar strain. As for hormone concentrations, both sleep-deprived Wistar and SHR showed lower testosterone concentrations than their respective controls. Sleep deprivation promoted an increase in concentrations of progesterone in Wistar rats, whereas no significant alterations were found after PSD in the SHR strain, which did not present enhancement in erectile responses. In order to explore the role of progesterone in the occurrence of genital reflexes, SHR were treated daily during the sleep deprivation period with progesterone; after the administration of this hormone and challenge with cocaine, we observed a significant increase in erectile events compared with the vehicle PSD SHR+cocaine group.

**Conclusion:**

Our data showed that the low frequency of genital reflexes found in SHR sleep deprived rats may be attributed to the lower concentrations of progesterone in these rats, based on the observation that progesterone replacement increased genital reflexes in this strain.

## Background

Hypertension, one of many common cardiovascular diseases in the developed world, is a noticeable risk factor for sexual dysfunction. A recent study reported erectile dysfunction in 35.2% of patients with essential hypertension compared with 14.1% of normotensive subjects [1]. Although it is commonly assumed that hypertension somehow predisposes men to erectile dysfunction, the precise pathogenic mechanisms involved have yet to be fully elucidated.

Spontaneously hypertensive rats (SHR) are widely used as animal models for essential hypertension since animals from this selectively bred strain gradually develop spontaneous arterial hypertension over a number of weeks. However, few studies have examined the effect of hypertension on sexual behavior in male rats and the results are still not conclusive. Clark and co-workers [2] reported that SHR showed significantly low incidence of penile erections (PE) in ex-copula tests. In the Wall et al. [3] study, SHR showed long mount latency and low mount frequency. On the whole, studies suggest impaired erectile capacity as evidenced by deficiency in penile reflex observed in SHR [3,4].

Recently our group has observed in several studies that paradoxical sleep deprivation (PSD) is a parameter that affects several behavioral responses [5-9] and has a positive effect on genital reflexes in Wistar rats [10-12]. This latter observation provided the initiative to examine other rat strains and compare the influence of PSD with or without cocaine administration on the occurrence of spontaneous genital reflexes among various strains. The great majority of our study has focused on the Wistar strain [11]. One possible factor involved in this stimulation is the alteration in neurotransmitter system sensitivity, as observed in dopamine supersensitivity [5], which leads to behavioral alterations in response to dopaminergic drugs following PSD. In this sense, drugs that stimulate dopamine neurotransmission may enhance sexual desire and arousal in human males [13] and stimulate mounts, intromissions, and ejaculation (EJ) in male rats [14]. And cocaine, a potent central nervous system stimulant, whose principal mechanism is believed to involve raising the availability of dopamine by blocking its reuptake [15], in association with PSD, enhances erections in rats more markedly than when these factors are isolated [10,16].

Therefore, our initial aim in this study was to investigate the influence of PSD associated with cocaine on genital reflexes in the SHR strain. The subsequent analysis of hormonal concentrations in PSD Wistar rats found significantly higher levels of progesterone than in control rats, prompting us to speculate that progesterone is an important hormonal factor that elicits genital reflexes in PSD males [17]. Corroborating this hypothesis, pretreatment with mifepristone (a progesterone antagonist) significantly reduced the percentage of rats displaying erections compared to control rats, and reduced erection frequency as well [12]. Since it has been shown that PSD can alter hormone concentrations, we have extended our previous findings by investigating whether the treatment with steroid hormones during PSD would influence on the genesis of genital reflexes in rats challenged with cocaine.

## Materials and methods

### Subjects

Adult (3–4 months) male Wistar rats were bred and raised in the animal facility of the Psychobiology Department-Universidade Federal de São Paulo (UNIFESP), and SHR were obtained from CEDEME- UNIFESP. The animals were maintained at 22°C with 12:12 h light-dark cycle (lights on at 0700 h) and allowed free access to food and water within standard polypropylene cages. All procedures used in the present study complied with the Guide for the Care and Use of Laboratory Animals. SHR are recognized as such when there is an increase in blood pressure and major alterations in both structural and mechanical vascular properties, which occurs at the age of approximately 12 weeks [18].

### Drug

Cocaine hydrochloride (Sigma Chemical Co., St. Louis, MO, USA) was mixed with sterile saline immediately before testing. This solution or saline (vehicle) was injected i.p. in a volume of 1 mL/kg. No animal received more than one experimental treatment. The 7 mg/kg dose was gauged on the basis that this dose was effective in inducing genital reflexes in sleep-deprived male rats [19].

### Paradoxical Sleep Deprivation (PSD)

The animals were submitted to PSD over a period of 96 h using the modified multiple platform method. We used this PSD period because it has been shown that most genital reflexes are produced during this span of time [16]. The rats were placed within a water tank (123 × 44 × 44 cm), containing 14 circular platforms (diameter 6.5 cm), placed in water up to 1 cm of their upper surface. The rats could thus move around inside the tank by jumping from one platform to another. When they reached the paradoxical phase of sleep, muscle atonia set in and they would fall into the water and awake. Throughout the study, the experimental room was maintained under controlled temperature (22 ± 1°C) and a 12 h light-dark cycle (lights on 0700 h-1900 h). Food and water were provided *ad libitum *by placing chow pellets and water bottles on a grid located on top of the tank. Tank water was changed everyday throughout the PSD period. Since we had found, in previous studies, that there were no genital reflexes in home-cage non-sleep deprived control animals, we used only cocaine-challenged PSD rats for this study, thus avoiding the use of a large number of animals.

### Genital reflexes

The rats were placed in experimental wire mesh cages (15 × 31 × 26 cm) containing neither water nor food, and behavioral observations were carried out from 0900 h to 1100 h in a temperature-controlled room. Trained observers blind to the experimental treatment group performed monitoring. PE was counted only when the rat displayed erection and bent down to lick its penis in full erection. EJ was scored by the number of ejaculatory plugs occurring with PE. The frequency of PE and EJ reflexes (total number of genital reflexes divided by number of rats) was assessed for 60 min.

### Hormonal analysis

For testosterone and progesterone analysis, blood was collected in glass tubes and centrifuged at 3,500 rpm for 10 minutes at room temperature, then frozen at -20°C until assayed. Intra-assay coefficients of variations are given in parentheses. Testosterone concentration (7.7%) and progesterone (9.8%) were measured by chemiluminescent enzyme immmunoassay (Advia Centaur, Bayer Corporation, Tarrytown, NY, USA). Duplicate serum aliquots were used.

### Experiment 1 – behavioral evaluation

**Figure 1:**
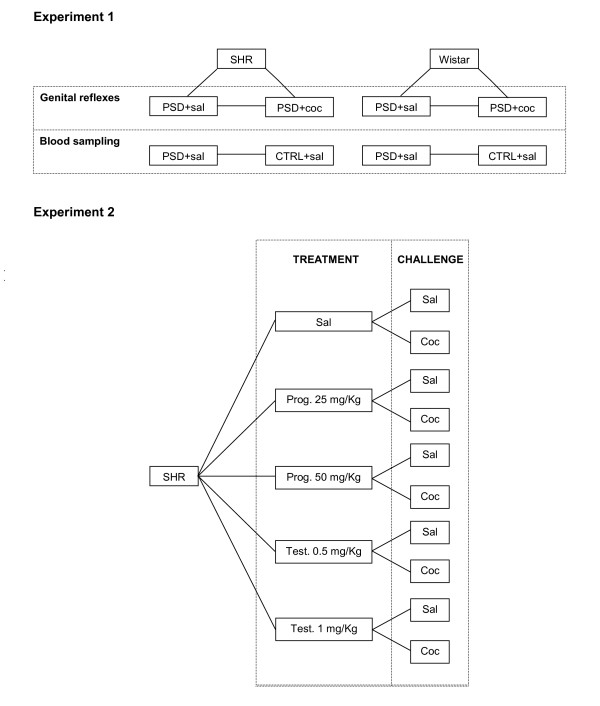
Schematic representation of the groups used in the present study. In Experiment 1, four groups of paradoxical sleep-deprived (PSD) animals each were studied: Wistar-saline; Wistar-cocaine; SHR-saline and SHR-cocaine; another set of home-cage control (CTRL) and PSD of SHR and Wistar strains were used for hormonal assessment. Experiment 2: hormone-treatment SHR groups were used: vehicle; 25 mg/kg or 50 mg/kg of progesterone; 0.5 mg/kg or 1 mg/kg of testosterone. After the PSD period each group was then subdivided into saline or cocaine-challenged groups.

When the experimental period ended, the PSD SHR and Wistar animals immediately were tested once for genital reflexes induced by an i.p. injection of cocaine (7 mg/kg) or saline. Thus, four groups of sleep-deprived animals each were studied: Wistar-saline; Wistar-cocaine; SHR-saline and SHR-cocaine (n = 10/group), as depicted in Figure 1.

To further examine the changes in hormonal concentrations after 96 hours of PSD and challenged with saline, we added non-sleep-deprived SHR and Wistar control groups (control-CTRL). Naive animals (n = 10/group) were sacrificed individually by decapitation with a minimum of disturbance in an adjacent room. Blood samples were immediately collected and stored individually.

### Experiment 2 – progesterone and testosterone treatment

Since after PSD progesterone concentrations were reduced in the SHR compared to the Wistar PSD group (see Results), we extended our study by investigating whether progesterone would play a facilitatory effect on the occurrence of genital reflexes in this strain. To determine the effects of hormone treatment on erection response, the SHR were distributed in three hormone-treatment groups (vehicle; progesterone 25 mg/kg and progesterone 50 mg/kg, n = 20 for each treatment regimen [10], and the hormonal injections were administered every morning during the sleep deprivation period. After this period, each of the hormonal treated groups were then subdivided into saline or cocaine-challenged groups (Figure 1).

Secondly, we added two groups treated with testosterone during the PSD period. The protocol adhered to that of the progesterone treated groups, thus the PSD-SHR males were treated with 0.5 mg/kg or 1 mg/kg of testosterone cipionate every day during the PSD period, totalizing 4 (s.c.) injections [10]. After this period each of the hormonal treated groups were then subdivided into saline or cocaine-challenged groups. To avoid using a large number of animals, and since our purpose was to enhance genital reflexes in SHR animals, only this strain was used for this set of experiments.

### Statistical analysis

In our statistical analysis of the numbers of animals displaying PE and EJ, the Chi-square test was used to assess inter-group differences. Frequency of genital reflexes and hormonal data were analyzed by two-way ANOVA test followed by Duncan test for comparison between treatment and vehicle groups. Values are expressed as mean ± SEM. The significance level was set at p < 0.05.

## Results

### Experiment 1 – behavioral evaluation

**Figure 2:**
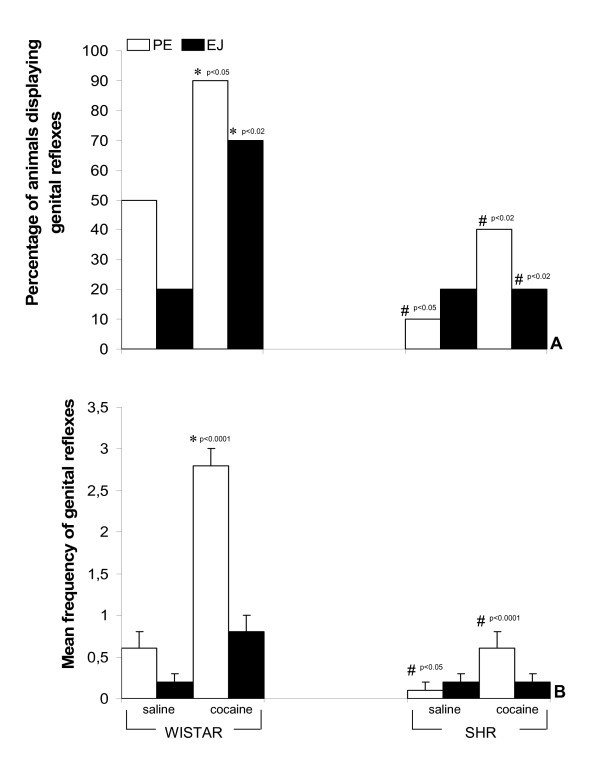
Effect of i.p. saline or i.p. cocaine (7 mg/kg) on genital reflexes in paradoxical sleep deprived Wistar and SHR rats. Panel A: Percentage of rats displaying genital reflexes and Panel B: Frequency of genital reflex events. *Different from same-strain (saline); ^#^Different from the respective group of Wistar strain. In Panel B, data are expressed as mean ± SEM for N = 10. Abbreviations: PE: penile erection; EJ: ejaculation; W: Wistar; SHR: spontaneously hypertensive rats.

As shown in Fig. 2A, PE was observed after saline injections in 50% of the PSD Wistar rats, and 2 of the 10 (20%) ejaculated. The 10% of SHR displaying PE was statistically different to the Wistar proportion (p < 0.05). Ejaculatory behavior was found for 20% of both SHR and Wistar rats. In addition, the results of the behavioral tests expressed as percentages of rats displaying PE show that PSD combined with cocaine in the Wistar strain induced PE in 90% of the Wistar rats; this effect was significantly reduced in SHR (p < 0.02) and also differed in relation to PSD Wistar saline rats (p < 0.05). The percentage of SHR ejaculating following cocaine injection was also significantly lower than in the Wistar-cocaine rats (p < 0.02). Wistar PSD+cocaine had significantly more EJ than its respective saline group (p < 0.02).

As for PE frequencies, two-way ANOVA revealed significant differences for strain [F(1,36) = 33.15; p < 0.000001], treatment [F(1,36) = 33.1; p < 0.00001] and interaction between the factors [F(1,36) = 15.6; p < 0.001] as shown in Fig. 2B. Post-hoc revealed that PSD+cocaine Wistar rats displayed significantly more PE during the behavioral test than the respective PSD+saline group (p < 0.0001). PE reflex was significantly decreased in SHR-PSD in relation to the Wistar rats (p < 0.05). The SHR-PSDThe frequency of EJ did not differ between the two strains (p > 0.05).

### Hormone concentrations

#### A) Testosterone

**Figure 3:**
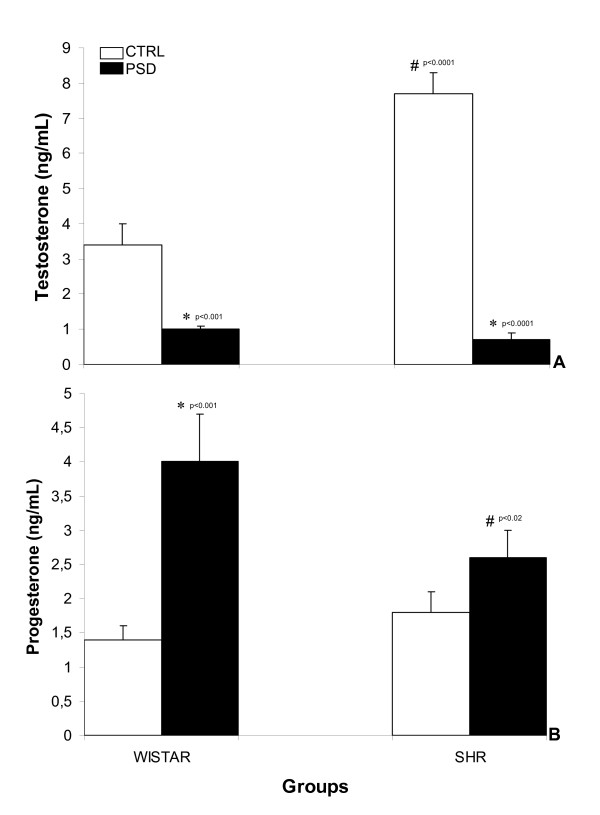
Mean ± SEM concentrations of serum testosterone (in ng/mL, panel A), and progesterone (in ng/mL, panel B) in control (CTRL) and paradoxical sleep deprivation (PSD) male rats. *Different from same-strain control rats; ^#^Different from the respective group of Wistar strain.

Analyzing the data with two-way ANOVA revealed significant group (control or PSD) [F(1,36) = 18.99, p < 0.0001] and strain (Wistar or SHR) [F(1,36) = 104.05, p < 0.00001] effects. Post-hoc testing indicated that Wistar PSD rats presented reduced testosterone concentrations when compared to respective control values (p < 0.001) and sleep-deprived SHR also differed from control rats (p < 0.0001). In addition, testosterone concentrations of control SHR were significantly higher than those of the Wistar control group (p < 0.0001) as shown in Fig. 3A.

#### B) Progesterone

Figure 3B shows the effects of PSD on progesterone concentrations in both strains. An ANOVA for progesterone concentrations revealed significant group effect [F(1,36) = 17.06; p < 0.0001]. Post-hoc testing indicated that progesterone concentrations were higher in Wistar PSD than compared to the control group (p < 0.001). A significant reduction in this hormone value was noted in the PSD-SHR in relation to the PSD-Wistar (p < 0.02).

### Experiment 2 – hormonal treatment

#### A) Progesterone

**Figure 4:**
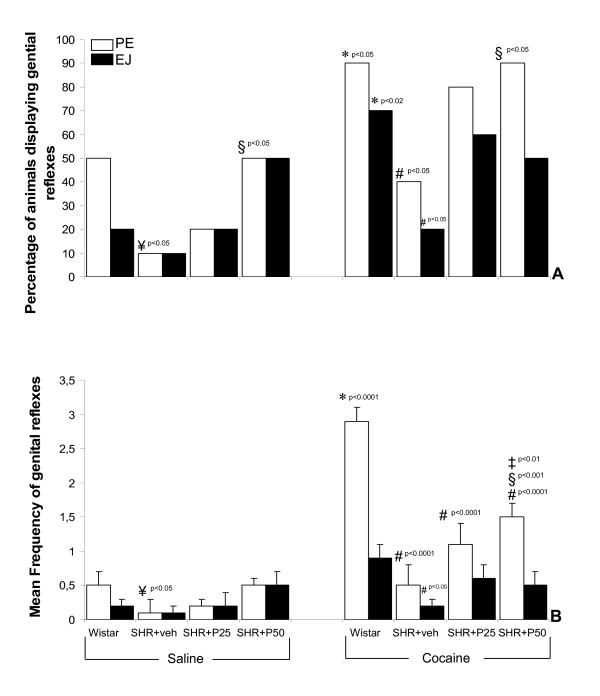
Effect of progesterone treatment (in mg/kg) on genital reflexes in paradoxical sleep deprived SHR. Panel A: Percentage of rats displaying genital reflexes and Panel B: Frequency of genital reflexes events. *Different from same-strain (saline); ^#^Different from Wistar strain (cocaine); ^¥^Different from Wistar strain (saline). ^§^Different from SHR+vehicle group; ^‡^Different from SHR+25 mg/kg+cocaine of progesterone. In Panel B, data are expressed as mean ± SEM for N = 10.

Figure 4 presents the percentage of PSD rats displaying genital reflexes and their frequencies for the Wistar and SHR strains of rat treated with vehicle or progesterone (25 and 50 mg/kg) during the PSD period.

As previously reported, PE after saline challenge injection was observed in 50% of PSD Wistar rats and 10% of the vehicle-SHR group (p < 0.05). On administering a 25 mg/kg progesterone dose there was no statistically significant difference in PE between SHR and Wistar rats, but with a dosage of 50 mg/kg, 50% of the SHR showed PE significantly different from that of the vehicle-SHR group (p < 0.05).

After cocaine injection, nine of the ten (90%) Wistar males displayed PE, a significantly higher percentage than the vehicle-SHR group (p < 0.05). The two progesterone doses increased the percentages of animals displaying erections to 80 and 90%. PE shown by the 25- and 50 mg/kg SHR groups did not differ from those in the Wistar rats. In addition, the 50 mg/kg progesterone SHR displayed significantly more erections than vehicle-SHR (p < 0.05). Furthermore, vehicle-SHR differed from the Wistar group in the percentage of animals ejaculating (p < 0.05).

As for PE frequencies (number of genital events displayed *per *group), the vehicle-SHR differed from Wistar-saline rats (p < 0.05), as depicted in Fig. 4B. After cocaine challenge, the PE frequencies observed in the SHR (vehicle and treated) groups were significantly lower than in the Wistar group (ps<0.0001). Moreover, the 50 mg/kg SHR differed from vehicle-SHR (p < 0.001) and 25 mg/kg SHR (p < 0.01), as shown in Fig. 4B. EJ frequencies observed in the vehicle-SHR differed from the Wistar group (p < 0.05).

#### B) Testosterone

**Figure 5:**
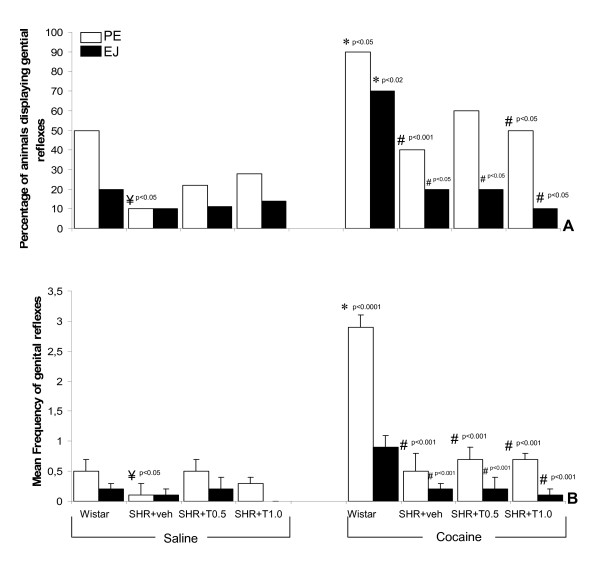
Effect of testosterone treatment (in mg/kg) on genital reflexes in paradoxical sleep deprived SHR. Panel A: Percentage of rats displaying genital reflexes and Panel B: Frequency of genital reflex events. *Different from same-strain (saline); ^#^Different from Wistar strain (cocaine); ^¥^Different from Wistar strain (saline). In Panel B, data are expressed as mean ± SEM for N = 10 (SHR+T 1 mg/kg+cocaine, N = 9).

The effects of testosterone treatment on the number of animals showing genital responses are presented in Fig. 5A. Neither testosterone dose produced a statistical increase in saline challenged male SHR compared to vehicle or Wistar groups, as seen in Fig. 5A. After cocaine injection, there was a significant decrease in the number of SHR given 1 mg/kg of testosterone displaying PE compared to Wistar animals (p < 0.05). No significant alterations were seen in the 0.5 mg/kg group. For EJ, testosterone treatment, regardless of dosage, induced a significant decrease in relation to the Wistar rats (p < 0.05).

As indicated in Fig. 5B, administration of testosterone did not have a significant effect on PE frequency in SHR compared to Wistar rats. After cocaine injection, the PE frequency was lower in the SHR+vehicle and SHR+testosterone (ps<0.001) rats compared to Wistar, as indicated by ANOVA followed by Duncan test [F(3,35) = 29.200; p < 0.00001]. The EJ frequency was also significantly lower in all SHR groups compared to Wistar (ps<0.001, ANOVA test [F(3,35) = 5.82; p < 0.002]) as shown in Fig. 5B.

## Discussion

This study was designed to determine whether spontaneous hypertension would influence genital reflexes in the sleep deprivation paradigm in the SHR strain compared with a normotensive strain. Our data showed that although PSD induced genital reflexes in the SHR, PE frequencies were significantly lower than in Wistar rats. Furthermore, we investigated the influence of a hypertension condition using a model that dissects the erectile response within the sleep deprivation paradigm in combination with a dopaminergic drug reputed to be a sexual stimulant.

Sleep deprivation is both common and critically relevant in our society and often occurs within a scenario of psychostimulant abuse, such as cocaine. Furthermore, these two factors are commonly related with heightened blood pressure [20,21]. Since factors that alter cardiovascular integrity would be expected to affect erectile response in human and animals, studies have demonstrated that hypertension is related to sexual dysfunction, in particular to erectile complaints. It has been shown that erectile tissue from SHR with established hypertension presented functional alterations [22]. Any factors modifying the basal corporal tone, the arterial inflow of blood to the corpora, the synthesis/release of neurogenic or endothelial nitric oxide, which is the principal peripheral proerectile neurotransmitter within the corpora and/or the veno-occlusive mechanism, are prime suspects for being involved in the pathophysiology of erectile dysfunction [23].

Sexual dysfunctions have drawn much attention in the last years [24-26]. As the pathogenesis of impotence associated with hypertension may be multifactorial, SHR strain models provide satisfactory investigation of this phenomenon. An increase in sympathetic function in the genitourinary organs, which may be causative of erectile dysfunction, has been reported in this strain [27]. However, despite the large body of knowledge regarding the neuropharmacology of sexual behavior in rats [14,28,29], little work has been done regarding sexual behavior in animal models of hypertension [2]. Clark and co-workers [2] reported SHR showing a reduced number of erections *per *test. On the whole, there is evidence that the current model of genital reflexes in PSD males corroborates with previous data on reflexive erection reported by Hård et al. [30] and Clark et al. [2,4]. More recently, a severe reduction in intracavernosal pressure plateau after electrical stimulation in SHR compared with normotensive rats has been documented [22].

In relation to hormone assays, testosterone concentrations were equivalent in SHR and Wistar-Kyoto (WKY) and more elevated than in Long Evans rats, a strain known to achieve a greater number of EJ and higher proportion of noncontact erections (see [31] and references therein) than other strains [32]. These findings suggest that alterations in testosterone concentrations may not be the underlying cause of strain differences in erectile function [11,17].

Concerning hormone alterations, the present results are in line with our previous studies reporting a marked reduction in testosterone in PSD rats whereas progesterone levels were increased significantly. Subnormal testosterone concentrations may contribute to sexual inadequacy in humans, which may affect actual or desired sexual relationships [33]. In rodents, however, testosterone *per se *does not seem to be the main factor accounting for genital reflexes observed in PSD rats since testosterone decreased in SHR males after a PSD period (Fig. 3A). The reduction of this hormone may be related to an increase of hypothalamus-pituitary-adrenal (HPA) axis activity, especially corticosterone concentrations, which are increased after sleep deprivation. Nevertheless, other classical stress procedures such as immobilization, swimming and footshock [34] or challenge with some illicit drugs in PSD were ineffective in inducing genital reflexes in rats [35]. Furthermore, the increased ACTH produced by stress may be responsible for an increase in progesterone levels [36]. Our findings have shown that progesterone is elevated in sleep-deprived rats and that this hormone is related to erectile frequency (for review [17]). There are data demonstrating that progesterone has a general suppressive effect on vascular smooth muscle contractility that seems physiologically more important than its additional, indirect vasoconstrictor effect (see [37] and references therein). Indeed, together with the involvement of this hormone in inducing erections in castrated rats [10], we tested different doses of antiprogestin mifepristone in PSD rats and found a significant reduction in PE for cocaine-injected rats compared to controls [12]. Similarly, it has been reported that supplementing progesterone in castrated male rats can trigger the full plethora of sexual behavior, even in the absence of other gonadal steroids [38]. Regarding the progesterone effects, it is well known that this hormone is synthesized in the adrenals, ovaries and also in the Leydig cells of testicles and exhibits both anti-androgenic and anti-mineralocorticoid effects. The possible central mechanism of progesterone may be related to structural and/or enzymatic changes that result from the interaction of the steroid with an intracellular receptor and subsequent activation of genomic regulated protein synthesis. Yet another possibility is its interaction with 5α-pregnan-3α-ol-20-one (allopregnanolone), the ring A-reduced metabolite of progesterone, with GABA-A receptor-gated chloride ion channel [39]. Still, the mechanisms through which sleep deprivation affects these hormones are currently being investigated.

For instance, synthetic progestins reduced REM/paradoxical sleep (PS) [40] and our data showed that mifepristone decreased the duration of PS episodes in male rats [12]. This suggests that progesterone must be involved in REM/PS regulation and that it may play a functional role in regulating genital mechanisms in males. Considering that PE is a characteristic phenomenon of REM/PS, the modulation of progesterone on erection as observed in our model could be attributed to its effects on REM/PS. The phenomenon of PS rebound is under strict regulation since there is a correlation between the duration of PS suppression and rebound duration [41]. Just as there is REM/PS rebound, events occurring during this phase of sleep, like erection, may also trigger a rebound phenomenon. Our observation of high erection frequency after PSD [16] prompted the hypothesis that erections occurring during sleep compensated for those the rodent did not display while awake.

On analyzing sleep architecture in the SHR strain, these rats showed less sleep time, poorer sleep quality [42], and shorter and less numerous PS episodes than the WKY strain [43]. Since the autonomic nervous system is closely related to blood pressure regulation, it would be worth investigating whether spontaneous hypertension is associated with a change in sleep patterns [43]. However, we still lack full comprehension of the mechanisms involved and interactions between factors.

In the current study, we speculated that progesterone concentrations might be higher in PSD-SHR but below the minimum required to trigger or facilitate PE. We therefore decided to treat the SHR animals with two different daily progesterone dosage levels during the PSD period. We found an increase in the percentage of SHR males displaying erection and the 50 mg/kg progesterone dose significantly increased PE. In the cocaine-challenged rats, this dose also led to an increase in the number of rats presenting erection compared to the vehicle group, and PE percentages caused by both progesterone dosages in SHR were similar to those in the Wistar strain. These observations suggest that genital reflexes in PSD rats may depend on progesterone secretion rather than being affected mainly by testosterone availability.

## Conclusion

In conclusion, the absence of high progesterone concentrations in sleep-deprived SHR may indicate a mechanism producing low PE frequency, especially in view of the fact that treatment with this hormone increased the percentage of rats displaying erection.
